# Winners or Losers? Two Academic Years in Experiences of COVID-19 Pandemic

**DOI:** 10.11621/pir.2024.0302

**Published:** 2024-09-15

**Authors:** Ivana M. Milovanović, Radenko M. Matić, Brigita Banjac, Saša Pišot

**Affiliations:** a *University of Novi Sad, Serbia*; b *Institute for Kinesiology Research, Science and Research Centre Koper, Koper, Slovenia*

**Keywords:** students, everyday practice, e-learning, perspective of the future, fear and loneliness, COVID-19 pandemic measures, public health

## Abstract

**Background.:**

The COVID-19 pandemic declared on March 11th, 2020, has had a substantial impact on the lives of all over the world. The student population, being one of the most vulnerable and substile ones, was forced to face specific unexpected circumstances for the first time in their lives.

**Objective.:**

In this paper, the authors explored the reflections of the COVID-19 experience and basic segments of everyday life of university students after the first academic year under pandemic measures and a follow-up year with their subjective perception of to what extent their lives have changed and how they were able to adapt to COVID-19 emergency measures.

**Design.:**

The field research was conducted among students in two European countries: Slovenia and Serbia. The qualitative semi-structured interviews with students (N = 20, 50% male) were executed in June-July 2020 and June-July 2021.

**Results.:**

The basic segments of students’ daily lives underwent significant changes, yet they successfully coped and adapted to the COVID-19 emergency measures. Notably, improvements were observed in study strategies, reducing fear and loneliness. Above all, the primary longing was for social contact and gathering with friends. In their private lives, they were more concerned about opportunities but on the other hand, they gained more free time for various non-academic activities. Finally, sports science students have proven better-coping mechanisms with extraordinary circumstances.

**Conclusion.:**

This study contributes to a deeper understanding of the changes in the daily lives of students during the first and second waves of the COVID-19 pandemic.

## Introduction

The origin of the COVID-19 pandemic is linked to Wuhan, China, and was first reported in December 2019. The virus rapidly spread globally, reaching Slovenia and Serbia in March 2020. During the first wave, Serbia immediately implement emergency measures (Cvetković et al., 2020). Conversely, Slovenia experienced a more significant impact during the second wave of the pandemic (Gorenjec et al., 2021). While these restrictive measures successfully curbed the spread of the virus, they had adverse effects on individuals (Hawryluck et al., 2004), including higher loneliness, poorer sleep quality, and mental health difficulties (Bakul & Heanoy, 2022; Dumciene & Pozeriene, 2022; Klanidhi et al., 2021).

The pandemic restricted various elements of the usual social actors daily life (Romero-Blanco et al., 2020) and has far-reaching consequences on education, health care, the economy, tourism, and relationships (Iyengar et al., 2020; Knight et al., 2021; Ochnik et al., 2021; Seyfi et al., 2023), resulting in uncertainty and numerous challenges (Van Tienoven et al., 2022). The shift to online classes during the lockdown is unsurprising led to a significant increase in sedentary behavior (Gallè et al., 2020). Given the established link between the level of physical activity and health-related quality of life (Meza-Miranda & Giménez, 2021), maintaining adequate PA plays an important role in preventing psychological problems (Elce et al., 2022; Tanir & Özmaden, 2018; Xiang et al., 2022).

The purpose of this study was to examine and analyze how students responded to changes in everyday life practices during the period of the COVID-19 pandemic restrictions. The researched domains were as follows: (1) Study: investigation of the to shift distance learning, adaptation in the subsequent study year; changes in residence for students in dormitories and rented apartments; study outcomes. (2) Feelings and Social Construct: exploring emotional responses, particularly fear and loneliness, within the context of the new circumstances of socialization. (3) Family life: examining the unplanned and intensive time spent with family members. (4) Personal life: assessing individual perspectives of the future, both personally and professionally.

This paper serves as a valuable contribution to addressing the gap in academic awareness concerning the potential consequences of emergency measures. Secondly, it sheds light on students’ adaptive and coping strategies developed to navigate the new circumstances of everyday life.

## Methods

This study employed a participants recruitment based on prior, larger-scale research with a mixed-method approach. The original study, conducted by a consortium (Pišot et al., 2022) included students from three European countries (Croatia, Slovenia, and Serbia) where the primary focus was to examine everyday life changes (daily habits, routines) during the first wave of COVID-19 pandemic emergency measures in 2020 (Pišot et al, 2022).

### Participants

The sample of the interviews consists of N = 20 (50% male, 50% female) university students with an average age of 21.86 years (SD ±2.06). Participants were drawn from different study programs and categorized into two groups. Group one includes sports sciences while group two encompasses other disciplines such as natural, technology, humanistic, and social sciences. The students represented the University of Novi Sad in Serbia as well as the Universities of Ljubljana, Primorska, and Alma Mater Europaea in Slovenia. See *Table 1* for the sample characteristics.

**Table 1:** Sample Characteristics

Variables	N	%
**Gender**		
Male	10	50
Female	10	50
**Study program groups**		
*Group 1 sports sciences (sports, kinesiology, physical education)*		
Male	4	
Female	5	
*Group 2 other sciences (natural, technical, humanistic, and social science)*		
Male	6	
Female	5	

### Procedure

Qualitative research was conducted among university students in Slovenia and Serbia to observe and analyze their responses to the changes in everyday life practices during the time of COVID-19 restrictions over two years (the first and second wave of the pandemic). The study included undergraduate and postgraduate programs and aimed to explore their experiences. A total of 40 semi-structured interviews were conducted, with 20 students, at two-time points; from June 10th to July 27th, 2020, and with a follow-up after a year from June 7 to July 20, 2021.

The interviews were structured around two central concepts: *(1) changes in everyday life routines and habits during COVID-19 measures focusing on aspects of sleeping and eating habits, physical activity, sedentary behavior, and screen time), and (2) the psycho-social impact of COVID-19 restrictive measures referred to personal relationship, feelings, coping with new study regimes, and perception of the future post COVID-19 experiences.*

Empirical data from the interviews, including transcriptions, annotations, codes, and node layouts, were made by the researcher and then stored and analyzed using the qualitative analysis software NVIVO 12. Researchers from Slovenia and Serbia followed and agreed-upon protocol for the initial code tree, tracking blocks of protocol questions, and any additional elements. The basic nodes for analysis included: “study strategies”, “feelings”, “socialization” and “personal life”. Detailed analysis of the data within these nodes involved examining study strategies — coping or adapting to new (e)learning; feelings — fear, loneliness, crampedness, etc.; investigation socialization in terms of what participants missed and lost from objective or subjective issues, family relationships; and assessing the perspective of the future, specifically how is related to career plans. The data analysis involved categorizing the impact of COVID-19 pandemic into sub-entries based on its duration and intensity (no impact, short-term impact, longer-term impact).

The conducted interviews carried out with a commitment to ethical standards, and without situations raising ethical concerns were encountered by the researchers. Anonymity was guaranteed to all participants, with each assigned a pseudonym (i.e., number) to ensure deidentification in the description of acquired data. Before participation, all interviewees willingly consented to the research by signing a consent form. Before the interviews, participants were provided with comprehensive information about the interview protocol, and the conditions under which acquired data would be utilized. The interviews were recorded using a dictation machine or smartphone application, and all recordings adhering to safety standards were securely stored in the researchers’ private databases.

The average length of the first wave of interviews was 23:37 minutes, with the shortest being 8:38 minutes and the longest being 50:53 minutes. During the second wave, the interviews were held between 20:28 and 44:39 minutes in Slovenia, as well as between 21:24 and 55:19 minutes in Serbia. The duration of the interviews was related to factors such as the type of interviewees (restrained-honest to completely honest), gender of the interviewees, year of study, and study program, but also the need and willingness of the interviewee to speak openly about his/her personal experience of the first and second wave emergency measures caused by the COVID-19 pandemic. All interviews (20 in the first wave were made in face-to-face form (offices or cafes), while in the second wave, 18 were recorded in person and two online, due to the incoming restriction measures of the COVID-19 pandemic. There were no significant differences observed between the online and in-person interviews. Participants were equally open and engaged in both formats, which we attribute to the semi-structured interview design and the comfortable environment provided by the researchers. This consistency suggests that the method of interview delivery did not influence the quality of the data collected.

## Results

### Study Strategies

The pandemic had a significant impact on the conventional academic routines of students. During the first wave of the pandemic, face-to-face classes were forced to switch to online learning, characterized by initial ambiguity, and lack of structure, and depended mainly on professors. Lectures were either shortened or canceled, and practical lectures were transformed into seminars in written form. While the shift to studying at home proved beneficial for some student, providing more flexibility and facilitating easier exam success, others encountered challenges such as difficulties in concentration, a higher stress level due to the online exam time constraints, and a lack of, cooperation opportunities with their peers.

The subsequent academic year presented a somewhat different scenario. During the second wave, professors adapted well and presented notable improvements. Classes were better prepared and organized often in a hybrid or online format.

*…they were very well prepared; you can see that they put that first period through and what didn’t work, and they were able to fix it in the second wave. It made the experience better; they were very approachable and responded promptly to emails on time. SL10*

*…all the practical work was postponed to May… and it was very reduced… SL5*

*It depends on the semester… winter semester was completed online, but when the summer semester started… classes started to be regularly held for students who are home, so they have practical work in the classrooms. SR2*

Even though the situation has improved, there are still some difficulties with distance learning, such as issues related to concentration, interaction, quality, or knowledge acquisition.

*Everything was online… all day at home. You wake up, open your computer, and sit in front of the screen until 6 pm. I had a hard time concentrating and it’s not the same as if the professor saw us, communicated with us. SL7*

*My biggest problem was online learning. Because I came in my fourth year and realized I didn’t know anything about those subjects. That I hadn’t absorbed the knowledge… SR1*

*It’s much harder to keep the focus on online teaching… SR4*

In addition to the negative aspects, students have also highlighted positive aspects. One notable advantage was that they improved ability to manage their time effectively better for study commitments. Then they can watch the recorded lectures again to better understand the lecture.

*So, I didn’t have any distractions and I could work on my work, I listened to the lecture and if I wasn’t interested, I did something else for the fax, so time wasn’t wasted… SL9*

*I think we even got a lot by recording our lectures and I think that helped us a lot later, when we were preparing for those exams. To listen again to something that we don’t understand, and that’s a big advantage. SR9*

*…I managed to prepare for some of the remaining exams and to graduate. SR8*

To summarize, students’ strategies improved, and their overall perception of academic life during the second year of the COVID-19 pandemic was more positive.

### Feelings

The pandemic, as an unexpected and unfamiliar phenomenon during the 2019/2020 academic year, triggered a range of emotions, including fear, anxiety, and stress. Among students, worries about potential health risks to their family members were a common concern. They also reported feeling discomfort and confusion due to the overwhelming information and imposed restrictions. Their longing for their pre-pandemic lives marked by unrestricted freedom of movement, was evident.

In the second interview round (2021), students emphasized the persistent lack of socialization and contact with family members, friends, and people in general.

*I was mostly worried that I couldn’t visit my parents, my sisters and brother, and my nephew, yes… we usually see each other at least once a month… the worst thing was that we didn’t know how long we wouldn’t be able to see each other. SL2*

*What I missed the most - all that was done in life… that we lacked contact with people, physical contact with people. SL5*

The university students found the constant changes to the restrictive measures against coronavirus infection confusing. They had to deal with psychological consequences such as fear or frustration, and the media only strengthened those negative feelings.

*It got my nerves because every week something was changing… because no one would convince me that after a week you could tell… whether a measure had an effect or not… and also people couldn’t get used to the measures so quickly. SL1*

*The time was so tight, that they were very weak… so they tightened again, and then weakened again. So, it mentally left, to be more accurate, impact on a lot of people… SR2*

*Fear for their own for this for that, and I think that the most was caused by this sensationalist reporting on all this I think it’s a disaster… SR8*

Among the various measures, the strictest ones, including restrictions between communities and lockdowns, were particularly challenging for the students to navigate.

*What bother were the restrictions on the municipalities… SL4*

Over time, however, the students became more familiar with the extraordinary circumstances and adapted better psychologically.

*For me, it was less stressful, less fearful for the future, less like panic attacks like the first wave, depression, and fatigue… SL1*

*…I would say that the view on the whole situation has changed, in the first wave… there was some fear present. No, I understand much more easily… it seems to me that everything is overblown… I still followed the measures. SL3*

The analysis also revealed that the students miss their freedom and opportunities to travel.

*On the other hand, I was longing to go anywhere. Freedom, no limits… SL5*

*We didn’t, so most of us didn’t have much opportunity to travel… SR5*

In summary, students, over time, decreased levels of worry, fear, and loneliness and showed improvement in their adaptation to the emergency measures caused by the COVID-19 pandemic. Notably, when compared to students from other disciplines (Group 2), those in sports science (Group 1) demonstrated generally more positive attitude.

### Socialization

The challenges posed by the first wave of the pandemic, including lockdown, social distancing protocols, and the shift to online education, significantly reduced face-to-face interaction. When students were asked about what they missed the most, a prevalent theme emerged — the loss of socialization, contact with friends, and public life. While some students reported minimal alterations to their daily routines, others indicated being forced to move out of the dormitory and return. Interestingly, the increased time spent with their parents and siblings in this period strengthened family relationships for some students.

The second round of interviews reaffirmed the crucial role of socialization and gatherings in the well-being of young people. Meaningful connections, positive relationships, and personal social interactions were identified as having numerous benefits for the individual.

*The hardest thing — quite honestly, I missed a bit of socializing with friends, I missed the weekend parties… SL3*

*For the first three months I didn’t see any of my friends… SL8*

*But most of all I lost that kind of contact with people… SR4*

Due to restrictive measures to maintain the connection between friends, the way of meeting and their activities had to be adapted. Face-to-face conversations were replaced by online meetings, games, and video or phone calls. As the measures have become less strict in the second year, students regained the opportunity to meet with friends in person again.

*The difference was that in the first one, I only saw a few (bubble), while in the second one, I saw more friends e.g., 1-2 times a month. SL3*

*With friends only practically via Zoom and video games … after they were split in, second semester I started hanging out… SL9*

*Just as it got back to normal, more and more I had the opportunity and options to go out with my friends from college, from high school, with everyone to see each other. SR10*

Social distancing measures forced students to return home, increase time spent indoors, and had more interaction with family members, which, in most cases, strengthened family relationships. On the other hand, some students reported prolonged periods without seeing their loved ones during this time.

*With the mother, normal communication, together at home. SL8*

*We’ve had a great relationship before, I just didn’t feel the need to tell my parents maybe everything. And now my parents have become like my friends to me, and they are versed in all my events. SR7*

*I had no contact with my parents at all because we were afraid that I don’t know… don’t transmit COVID… and haven’t seen my parents for a very long time… SR8*

In general, the imposed restrictive measures resulted in isolation and a pervasive sense of loneliness among the students.

### Personal life

In general, the students reported in the first interviews that their lives had slowed down, becoming less structured. Their familiar habits and routines were disrupted, and the new normal was characterized by monotony and undefined obligations. Consequently, they had more free time for non-academic activities, such as pursuing a new hobby, engaging in regular physical activity, spending quality time with family members, and contemplating future priorities and careers. Despite awareness of the potential for working online, the majority of students expressed concerns about diminished job opportunities and finances. Their summer plans underwent alterations, with social events (sports, concerts, or festivals) being postponed or canceled. Due to the restrictive measures, students refrained from planning trips or vacations focusing primarily on studies or writing their thesis. However, in some cases, the pandemic had no impact on work or career.

The second interview highlighted an improvement in students’ daily lives although they continue to face challenges.

*I remember there were two weeks of university and then everything stopped again. SL8*

*…if I had to describe the whole year, I could even describe it in a few words. I’d call it a big change. SR1*

Compared to the first wave of the pandemic, maintaining daily habits and routines was easier for students. With fewer study obligations than pre-pandemic times, students find themselves more engaged in healthier behaviors, hobby activities, or dedicating their time to personal development.

*No there were no changes, I kept my biorhythm quite normal… SL1*

*I’ve started working on myself a lot over the past year. SR2*

*When it comes to sleep, I slept a lot more regularly… SR9*

*Physical activity didn’t suffer so much because it’s something I love to do, so I’ve always found a way to do it. SR4*

*I did a lot of sport… I’ve found that it relaxes me. A new hobby… I read more books than I used to. SL7*

When comparing sports science students and those in other study programs, it became apparent that a higher proportion of sports science students tended to maintain healthier habits (*e.g*., regular exercise, balanced diet) across the pandemic waves, where this trend was less present in the other group of students.

Students reported varied perspectives on their future and careers. While some emphasized positive effects including new job opportunities arising of the pandemic, a significant number expressed concerns and worries about their prospects.

*The problem was to get a student job… also from a financial point of view… SL4*

*At the beginning of this year, I started working in a sports school because I realized that I just had time for something like that. SRB4*

Given that the pandemic lasted over two years by the time of the second interview, it is not surprising that it could had various consequences for some students.

*There might be consequences on the labor market because there will be a generation that has no skills… no experience, no laboratory exercises… SL10*

*In the last two months, things have returned to normal. SR9*

*Maybe I appreciate some things more. SR9*

Based on the results of this study, it can be concluded that the basic segments of the students’ daily routines have undergone changes, and they demonstrated the ability to adapt to the emergency measures imposed by the COVID-19 pandemic.

## Discussion

This study focused on the students’ reflection of their basic segments on everyday life practices during two key periods: the first academic year under pandemic measures (June-July 20) and the subsequent year (June-July 21). Students provide a subjective perception of the changes they experienced in their lives and discussed their strategies for adapting to the challenges posed by the COVID-19 emergency measures.

One of the preventive measures implemented during pandemic was the switch from face-to-face to online learning (Wang & Zhao, 2020). Initially, the lack of use of technology and technical issues such as internet connection were a problem (Suryaman et al., 2020). In the second academic year of the pandemic, there was notable improvement as professors and classes were better prepared and organized for online learning. Nevertheless, despite these achievements, the online format still differed from in-person classes with persisting issues like lack of motivation, concentration, reduced quality, and limited social interactions with professors and peers. On a positive note, students were happy with recorded lectures and appreciated the increased flexibility in organizing their time.

Even before the first wave of the pandemic, university students were prone to high levels of anxiety and stress (Vigouroux et al., 2021). As the pandemic rapidly spread and persisted, their stress levels, anxiety, and loneliness intensified (Elmer et al., 2020). Students began to concern for the well-being of their family and friends, and fears of infecting with the coronavirus also become prominent (Chesser et al., 2020). On the other hand, some students reported not feeling significantly affected by the circumstances (Knight et al., 2021). The influx of information, coupled with inconsistent measures and travel restrictions contributed to pervasive sense of uncertainty and negative emotions, including anxiety, frustration and stress).

The impact of reduced social interaction was observed in both waves of the pandemics student faced restriction on their social life. Social distancing measures led to prolonged periods of separation from peers and friends and other social opportunities (Crăciun, 2024). Coping with this situation, students found ways to maintain contact, and as time progressed and measures eased, they gradually returned to their usual social habits, meeting in person. Notably, as they become more familiar with to the ongoing pandemic, students reported improved mental health experiencing lower levels of fear, frustration and loneliness. Another important consequence was the enforced reallocation of students from campus (Conrad et al., 2021). Interviews also revealed that they had to move back home, leading to increased time spent with family members and strengthening their interpersonal relationships. However, it is important to note that some students stated not seeing their family members for an extended period during this time.

The restrictive measures during the pandemic prompted individuals to yearn for the freedom to travel, having holidays, or, simply enjoy a coffee with friends. These circumstances led people to reassess their priorities, goals, and careers aspirations. Prolonged restrictions heightened concerns about the future among students (Elmer et al., 2020). Even though some employment opportunities have arisen such as online jobs or positions in healthcare, these were fewer compered pre-pandemic time. In addition, students’ contemplation their future plans found themselves canceling or postponing vacations and trips. On a positive note, the reduced academic obligations provided students with more time for non-academic commitments, hobby activities, and the cultivation of healthier routines. Overall, exhibited increased resilience and coping abilities during the second academy year (2020/2021).

Physical activity plays an important role in improving mood and mental health (Liverpool et al., 2023). Given that sports science students were physically active during the COVID-19 pandemic (Özkan et al., 2021), it is not surprising that they reported better coping mechanisms and responded positively to the complex situation of the pandemic (Banjac et al., 2023). Our findings align with existing literature, indication that sport science students, in comparison to their counterparts in other study programs, performed greater adaptability and resilience, they tend to maintain their daily habits and routines in an altered form (*e.g*., continue to exercise indoors) throughout the COVID-19 pandemic period.

University students found themselves navigating a complex landscape during the pandemic, experiencing both victories and defeats. Losers in this scenario encountered disruptions across multiple facets of their lives, spanning study strategies, social interactions, habits, and considerations about their future. The pervasive feeling of uncertainty loomed, with the awareness that everything could undergo abrupt changes. Despite these challenges, students — winners showed resilience by demonstrating an ability to cope with the unusual circumstances posed by the pandemic, adapting their everyday life practices. However, amidst this adaptation, they lamented the loss of their familiar and traditional student life, underscoring the multifaceted impact of the pandemic on their collegiate experience.

## Conclusion

The research provides valuable insights into the everyday lives of the student population, offering a better understanding of their perception of “new normal” and how young, predominantly healthy individuals cope with emergency measures imposed by the COVID-19 pandemic. Various studies highlight negative consequences resulting from these measures, such as poorer mental health with post-traumatic stress symptoms, avoidance behavior, anger, fears, frustrations, boredom, stigma, lack of supplies, lack of adequate information, financial loss (Brooks et al., 2020; Hawryluck et al., 2004; Kornienko & Rudnova, 2023; Reynolds et al., 2008; Wang & Zhao, 2020). Specifically, loneliness emerged as a particularly substantial consequence (Werner et al., 2021), therefore, social support is crucial in these circumstances (Eigege & Kennedy, 2021).

From the educational perspective, research indicates some negative impacts or reveals some of the challenges and obstacles experienced by students in online or e-learning refers to limited communication, outreach, and extended screening time (Radu et al., 2020; Suryaman et al., 2020). The unexpected onset of the COVID-19 pandemic prompted universities to act fast (Strielkowski, 2020), causing uncertainty among students for employability and career planning (Capone et al., 2021), as the university system was firstly shortly closed and then shift to online learning (Alsoufi et al., 2020). Consequently, students experienced uncertainty. If we consider the type of the study, the sport science students adapted better (Pišot et al., 2022) with a moderate level of uncertainty (Ulukan, 2021).

In conclusion, the basic elements of students’ daily lives have changed showing their resilience and adaptability to the challenges posed by the COVID-19 emergency measures. Winners in this scenario improved their study strategies, experiencing reduce fear and loneliness and gained more free time for various non-academic activities. Remarkably, sport science students showed better coping mechanism, drawing on their prior knowledge and experiences of health-related practice in daily routines.

Conversely, the losers were marked by a continued longing for social contact and gathering with friends. Students expressed concerns about future job opportunities which became a prominent aspect of their personal life.

Overall, this not only addresses the gap of academic awareness of the potential consequences of emergency measures but also provides a deeper understanding the transformations in students’ daily lives during the first and second wave of the COVID-19 pandemic. It offers valuable insights into their adaptive strategies in navigating these unprecedented circumstances.

## Limitations

This study examined the experience and basic segments of everyday life of university students after the first and second waves of the COVID-19 pandemic. Further research could enhance the study by incorporating a larger and more diverse sample size for interviews, including participants from different countries. Continual investigation into the evolving dynamics of students’ lives in the context of the past impact of the pandemic can contribute to a more comprehensive understanding of their adaptive processes and the long-term effects of such disruptions.
